# 

**Published:** 1852-10

**Authors:** 


### RECEIPTS

### FOR THE JOURNAL DURING THE MONTH OF SEPTEMBER

| ILLINOIS. | | WISCONSIN. | |
|---|---|---|---|
| Dr. G. W. Albin, - - - | 2 00 | Dr. John L. Scofield, - - | 2 00 |
| " J. J. Lischer, - - - | 2 00 | " S. C. Johnson, - - - | 2 00 |
| " J. H. Wilkinson, - - - | 2 00 | " G. W. Lee, - - - | 2 00 |
| " J. Bennett, - - - | 2 00 | " R. J. Tilbits, - - - | 2 00 |
| " E. G. Mygatt, - - - | 2 00 | " J. M. Ball, - - - | 2 00 |
| " L Fuller, - - - | 1 00 | MICHIGAN. | |
| INDIANA. | | Dr. J. W. Joyce, - - - | 2 00 |
| Dr. A. V. Talbert, - - - | 2 00 | | |
| " A. Davisson, - - - | 2 00 | | |
| " J. P. Tucker, - - - | 2 00 | | |

### PROSPECTUS

OF THE NEW SERIES OF THE

### NORTH-WESTERN

### MEDICAL AND SURGICAL JOURNAL.

EDITED BY

**W. B. HERRICK, M.D.,**

*Professor of Anatomy and Physiology in Rush Medical College; Surgeon to the U.S. Marine Hospital; and one of the Surgeons of the Ill. General Hospital.*

ASSISTED BY

**H. A. JOHNSON, M.D.**

---

THE NORTH-WESTERN MEDICAL AND SURGICAL JOURNAL will be published hereafter, on the first of each month, beginning in May, in numbers, each containing, exclusive of advertisements, Forty-eight pages, closely printed, with new type ordered expressly for this work; thus presenting at the end of the year a volume of nearly Six Hundred pages.

The change from a bi-monthly to a monthly Journal, has been made in accordance with the expressed wishes of numerous subscribers.

The editor, with his associate, Dr. JOHNSON, who will devote his time exclusively to the Journal, will endeavor, in addition to a full and complete Domestic Summary, to present numerous extracts from English, and translations from French and German Medical periodicals.

In addition to the Communications from Contributors, whose favors, it is hoped, will be continued, the Original Department will contain, from time to time, reports of Cases treated, and of Observations made, in the two Hospitals, now fully organized in Chicago.

---

### TERMS:

**TWO DOLLARS PER ANNUM, IN ADVANCE.**

---

As an inducement to Physicians and others to act as Agents, in extending the circulation of the Journal,

| | |
|---|---|
| Three copies will be furnished to new subscribers for | $5 00 |
| Five " " " | 8 00 |
| Seven " " " | 10 00 |

---

### TERMS OF ADVERTISING.

| | |
|---|---|
| One page, One year ............$20,00 | One page, single insertion........$5,00 |
| One-half page, One year, ...... 12,00 | One-half page, single insertion.... 3,00 |

---

☞ *Letters must be post-paid, and directed to the* "NORTH-WESTERN MEDICAL AND SURGICAL JOURNAL, *Chicago, Ill.*" *A list of receipts will appear in each No.*

